# Racial and socioeconomic inequities in breast cancer screening before and during the COVID-19 pandemic: analysis of two cohorts of women 50 years + 

**DOI:** 10.1007/s12282-022-01352-2

**Published:** 2022-04-02

**Authors:** Pablo Monsivais, Solmaz Amiri, Jeanne Robison, Chaya Pflugeisen, Gordon Kordas, Ofer Amram

**Affiliations:** 1 Department of Nutrition and Exercise Physiology, Elson S. Floyd College of Medicine, Washington State University, 412 E Spokane Falls Blvd, Spokane, WA 99202 USA; 2 Institute for Research and Education To Advance Community Health, Elson S. Floyd College of Medicine, Washington State University, Seattle, WA USA; 3 MultiCare Institute for Research and Innovation, Tacoma, WA 984145 USA; 4 MultiCare Deaconess Cancer and Blood Specialty Centers, Spokane, WA 99204 USA; 5 Department of Medical Education and Clinical Sciences, Elson S. Floyd College of Medicine, Washington State University, Spokane, WA USA; 6 Paul G, Allen School for Global Animal Health, Washington State University, Pullman, WA 99164 USA

**Keywords:** Cancer prevention, Screening mammography, Race and ethnicity, Socioeconomic status, Health equity, Covid-19 pandemic, Health services

## Abstract

**Background:**

Routine screening mammography at two-year intervals is widely recommended for the prevention and early detection of breast cancer for women who are 50 years + . Racial and other sociodemographic inequities in routine cancer screening are well-documented, but less is known about how these long-standing inequities were impacted by the disruption in health services during the COVID-19 pandemic. Early in the pandemic, cancer screening and other prevention services were suspended or delayed, and these disruptions may have had to disproportionate impact on some sociodemographic groups. We tested the hypothesis that inequities in screening mammography widened during the pandemic.

**Methods:**

A secondary analysis of patient data from a large state-wide, non-profit healthcare system in Washington State. Analyses were based on two mutually exclusive cohorts of women 50 years or older. The first cohort (*n* = 18,197) were those women screened in 2017 who would have been due for repeat screening in 2019 (prior to the pandemic’s onset). The second cohort (*n* = 16,391) were women screened in 2018 due in 2020. Explanatory variables were obtained from patient records and included race/ethnicity, age, rural or urban residence, and insurance type. Multivariable logistic regression models estimated odds of two-year screening for each cohort separately. Combining both cohorts, interaction models were used to test for differences in inequities before and during the pandemic.

**Results:**

Significant sociodemographic differences in screening were confirmed during the pandemic, but these were similar to those that existed prior. Based on interaction models, women using Medicaid insurance and of Asian race experienced significantly steeper declines in screening than privately insured and white women (Odds ratios [95% CI] of 0.74 [0.58–0.95] and 0.76 [0.59–0.97] for Medicaid and Asian race, respectively). All other sociodemographic inequities in screening during 2020 were not significantly different from those in 2019.

**Conclusions:**

Our findings confirm inequities for screening mammograms during the first year of the COVID-19 pandemic and provide evidence that these largely reflect the inequities in screening that were present before the pandemic. Policies and interventions to tackle long-standing inequities in use of preventive services may help ensure continuity of care for all, but especially for racial and ethnic minorities and the socioeconomically disadvantaged.

## Background

Breast cancer remains the most common cancer among women, with an average annual rate of 125 cases per 100,000 in the United States [1]. However, over the last 30 years, deaths from breast cancer have declined to an average annual rate of 20 per 100,000 [2] and a 10-year survival rate of 84% [3]. Increasing survival rates have been attributed to more effective treatment options, but also to earlier detection among women who participate in routine breast cancer screening.

While a small percentage of women with above average risk may be considered for screening before age 50, both government agencies [4, 5] and key medical societies [6] are in agreement that screening mammograms should be offered to all women by age 50. There remains debate among these groups whether to recommend yearly versus every two-year screening, but there is still consensus that bi-annual screening is the minimum health maintenance goal among women with average risk.

Despite consistent breast screening guidelines, there are existing inequities in the use of these secondary preventive services which, if used effectively, have the potential to detect breast cancers in the earliest stages. For example, 73% of non-Hispanic white women aged 50–74 reported having undergone mammographic screening in the past two years, compared to 66% of American Indian/Alaska native women [3]. Similarly, 75% of women with health insurance coverage reported having undergone screening compared to 39% of women without insurance [3].

Such existing inequities in screening utilization may have been amplified by the coronavirus pandemic, which reduced access to health services [7] potentially resulting in adverse impacts on economic insecurity [8] and other social determinants of health [9]. Although the impact of the pandemic-related closures and stay-at-home orders have been described for screening services overall [10–12], including breast screening [13, 14], less is known about breast cancer screening utilization among different sociodemographic groups during the pandemic.

Using individual-level patient data from a large health care network, we tested the hypothesis that the COVID-19 pandemic amplified inequities in breast screening utilization among women aged 50 years and older, who were patients in a large health care network in Washington state, USA. We examined data from women who were eligible for radiographic screening (mammography) on a biannual basis, had a screening in 2018, and would have been due for a repeat regular screening in 2020, in the midst of the pandemic. These women were compared to women presenting for screening in 2017 who would have been due in 2019 and analyses explored whether sociodemographic gaps in two-year return screening differed between the two cohorts of women.

## Methods

### Data source

This secondary data analysis included completed screening mammograms within MultiCare health system, a large state-wide community non-profit healthcare system in Washington State. The health care delivery system includes over 230 primary care, specialty care and urgent care clinics, and eight hospitals across Washington State. The study protocol was approved by the Institutional Review Board of MultiCare, the data holder.

### Population sample

This study was an analysis of two mutually-exclusive cohorts of women selected based on inclusion criteria in either 2017 or 2018. The first cohort were those women meeting inclusion criteria screened in 2017 who would have been due for repeat screening in 2019 (prior to the pandemic’s onset). The second cohort were women screened in 2018 due again in 2020. The inclusion criteria required women in the sample be 50 years or older who had completed a screening mammogram between January 1 and December 31 of the two base years of each cohort (2017 or 2018) but did not have a mammogram in the following year (2018 or 2019, respectively).

### Outcome variable

The outcome variable was completion of a two-year follow-up screening mammogram during a 12 month period within the health system (yes vs. no). The odds of two-year follow-up screenings in 2020 were compared to the same interval in 2019, a period prior to the pandemic. Together with our inclusion criteria, this variable represents women 50 years or older who were screened according to the guidelines for breast cancer screening [6] and those who became technically over-due for breast cancer screening during the first year of the Covid 19 pandemic.

### Explanatory variables: sociodemographic characteristics

Sociodemographic characteristics of patients were collected from patients' electronic health records. We categorized age using three groups (50–64 years, 65–69 years, and 70 years and older) corresponding to age thresholds for routine screening recommendations. Insurance status was collapsed into commercial, government (Tricare, Champva, or Worker’s Comp), Medicaid, Medicare, self-pay, or unknown. Race or ethnicity was categorized into White, American Indian and Alaska Native (AIAN), Asian, Black, Hispanic, Multi-racial, Native Hawaiian or other Pacific Islander (NHOPI), or Unknown. Rural or urban residence was derived using urban–rural communing area (RUCA) codes at the ZIP code level [15, 16]. Residential ZIP codes with RUCA primary codes of 1–3 were classified as urban areas and those with codes of 4–10 were classified as rural.

### Statistical analysis

Descriptive statistics included frequency distributions and percentages. For each cohort, two separate, multivariable, generalized linear models (GLMs) with binary logistic link functions and all sociodemographic variables included as fixed effects were fit to determine adjusted odds of screening at two years after initial screening.

In order test whether screening inequities that existed prior to the pandemic differed during the pandemic, we evaluated logistic regression models that included interaction terms between each of the sociodemographic variables (race/ethnicity, insurance type, rurality, and age category) and cohort variable (indicating those who were scheduled to be screened in 2019 and those who were scheduled to be screened in 2020). Fitting separate interaction models for each explanatory variable allowed us to assess the change in screening relative to the variable reference group during the pandemic. Simply, whether the change in screening for a particular sociodemographic group during the pandemic is significantly different relative to what it was pre pandemic when compared to the reference group. For all models, associations were presented as odds ratios (ORs) with 95% confidence intervals (CIs) and *p*-values at 0.05 were considered significant. Interactions effects are ratios of odds ratios. All analyses were performed in R version 3.6 and models were fit using the base R functions. Profile plots were generated using the interaction package in R.

## Results

Our 2018 cohort included 16,391 women 50 years and older who underwent mammography in 2018, but not in 2019 (Table 1). Our 2017 cohort included 18,197 women who met inclusion criteria. Nearly half of the sample was between ages 50 and 64 and 97% resided in urban areas. Whites made the majority of the sample (81%), with Asian, Black, and multi-racial women being the next largest groups (5, 4, and 3% respectively). The major types of insurance coverage were commercial (36%) and Medicare (39%). Of the 2018 cohort, approximately 27% were also screened in 2020, and the demographic profile of these patients was slightly different, with larger percentages of older women, whites, urban residents, and those insured through commercial and Medicare policies.

**Table 1:** Characteristics of women age 50 + Y who had breast cancer screening in 2018 and not in 2019, overall and stratified by whether they had a subsequent screening in 2020 (*n* = 16,392)

Characteristics	2017 cohort	Rescreened at 2019 (*n* (%))	2018 cohort	Rescreened at 2020 (*n* (%))
Overall	18,197	6660 (36.6%)	16,391	4438 (27.0%)
Age				
50–64	8739	3117 (35.7%)	8365	2261 (27%)
65–69	3388	1341 (39.6%)	2858	842 (29.5%)
70 +	6052	2202 (36.4%)	5168	1335 (25.8%)
Insurance				
Commercial	6286	2374 (37.8%)	5948	1755 (29.5%)
Government	211	64 (30.3%)	170	36 (21.2%)
Medicaid	959	256 (26.7%)	902	142 (15.7%)
Medicare	7048	2633 (37.4%)	6032	1727 (28.6%)
Selfpay^a^	272	44 (16.2%)	141	25 (17.7%)
Unknown	3404	1298 (38.1%)	3199	653 (20.4%)
Geography				
Urban	17,791	6561 (36.9%)	16,072	4388 (27.3%)
Rural	372	94 (25.3%)	310	50 (16.1%)
Race/ethnicity				
White	14,689	5486 (37.3%)	13,191	3702 (28.1%)
AIAN^b^	73	11 (15.1%)	58	13 (22.4%)
Asian	903	368 (40.8%)	920	223 (24.2%)
Black	852	320 (37.6%)	900	207 (23%)
Hispanic	537	185 (34.5%)	449	105 (23.4%)
MultiRacial	688	224 (32.6%)	649	151 (23.3%)
NHOPI^c^	139	45 (32.4%)	135	26 (19.3%)
Unknown	298	21 (7%)	99	11 (11.1%)

^a^Patients who paid for the procedure out-of-pocket and typically lacked health insurance

^b^American Indian or Alaska Native

^c^Native Hawaiian or Pacific Islander

In our multivariable model examining only the 2018–2020 cohort, we identified sociodemographic factors associated with reduced odds of screening in 2020 (Table 2, right). In particular, women who were aged 70 + showed reduced odds of being screened in 2020, compared to women 50–64 (OR, 0.83). Relative to whites, all other racial and ethnic groups showed lower odds ratios, ranging from 0.61 to 0.9. Asian, Black, and multi-racial women showed significantly reduced odds of being screened in 2020 (ORs, 0.84, 0.80, and 0.77, respectively). Compared to women with commercial insurance, those who were on Medicaid and who self-paid for care had reduced odds of having follow-up screening (ORs, 0.43 and 0.53, respectively). Finally, rural women showed substantially reduced odds of being re-screened compared to urban women (OR, 0.50) in 2020. Sociodemographic inequities in repeat screening at two years were similar for women due in 2019, prior to the pandemic (Table 2, left). With a few exceptions, ORs were similar or closer to 1 in 2019 compared to 2020, suggesting that the inequities remained or widened during the pandemic.

**Table 2:** Odds ratios (OR) from two multivariable-adjusted logistic regression models assessing odds of screening in 2019 for women last screened in 2017 (left), and odds of screening in 2020 for women last screened in 2018 (right)

Variable	2017 Cohort, Adjusted odds of screening in 2019		2018 Cohort, Adjusted odds of screeining in 2020	
	OR (95% CI)	*p*-value	OR (95% CI)	*p*-value
Age				
50–64 (Ref)	–		–	
65–69	1.14 (1.03–1.27)	0.015	1.03 (0.91–1.16)	0.676
70 +	0.94 (0.84–1.05)	0.293	0.81 (0.71–0.99)	0.001
Insurance				
Commercial (Ref)	–		–	
Government	0.74 (0.55–1.00)	0.053	0.66 (0.45–0.95)	0.03
Medicaid	0.60 (0.52–0.70)	< 0.001	0.46 (0.38–0.56)	< 0.001
Medicare	1.00 (0.91–1.11)	0.938	1.08 (0.96–1.22)	0.192
Self-pay	0.35 (0.25–0.48)	< 0.001	0.53 (0.31–0.63)	0.005
Geography				
Urban (Ref)	–		–	
Rural	0.57 (0.44 – 0.74)	< 0.001	0.50 (0.35 – 0.69)	< 0.001
Race/ethnicity				
White (Ref)				
AIAN	0.30 (0.14–0.58)	0.001	0.64 (0.30–1.23)	0.294
Asian	1.19 (1.01–1.40)	0.037	0.91 (0.75–1.09)	0.303
Black	1.05 (0.89–1.23)	0.561	0.84 (0.70–1.00)	0.053
Hispanic	0.87 (0.69–1.09)	0.231	0.81 (0.61–1.06)	0.139
Multi-racial	0.86 (0.72–1.03)	0.105	0.81 (0.65–0.99)	0.045
NHOPI	0.97 (0.64–1.44)	0.885	0.66 (0.39–1.06)	0.099
Unknown	0.29 (0.17–0.45)	< 0.001	0.42 (0.20–0.78)	0.010

All ORs are mutually-adjusted in each model

**Fig. 1:**
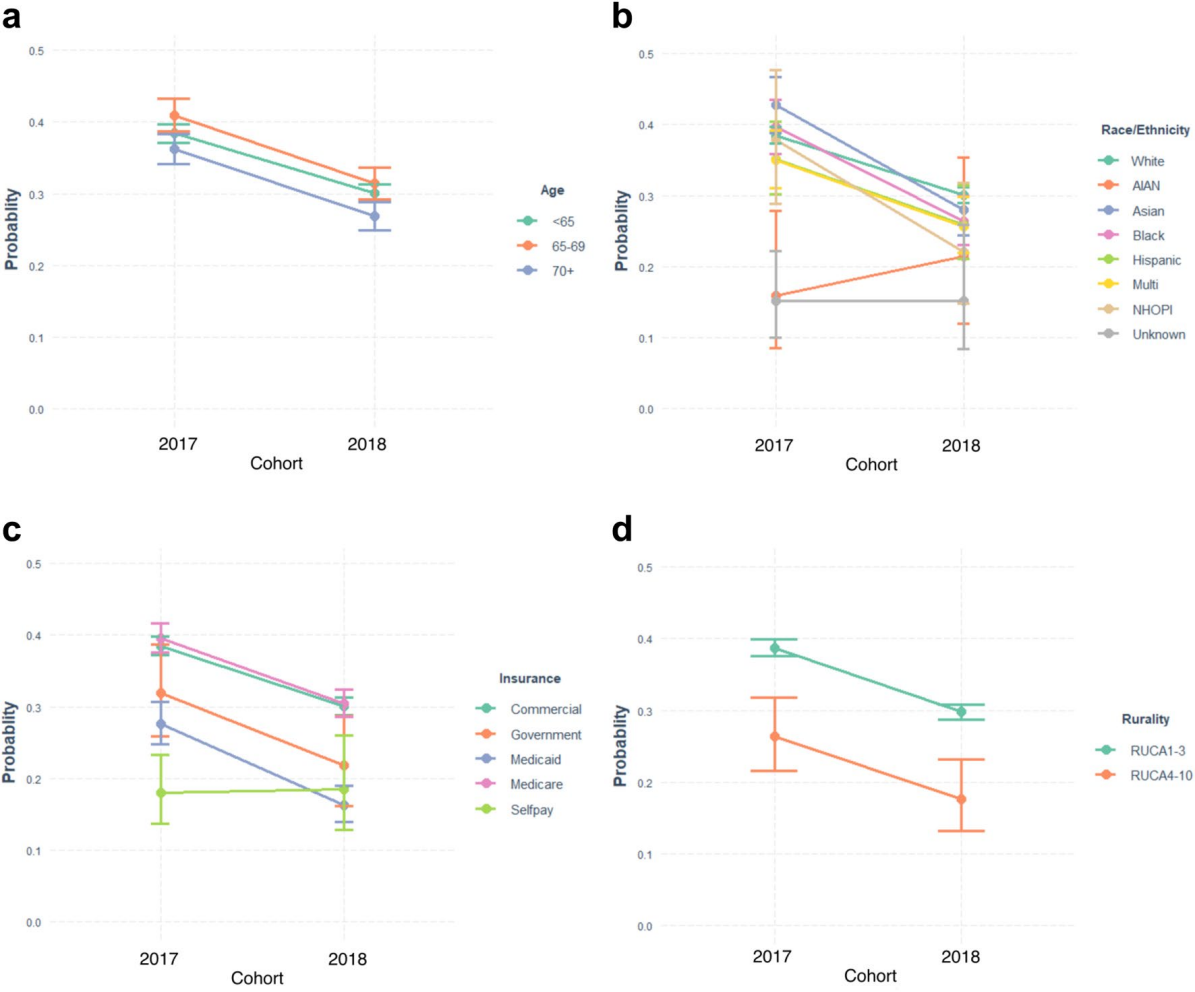
Profile plots of predicted probabilities for screening at two years for two cohorts of WA state women who would have been due for routine screening in 2019 and 2020. Stratified by age group (panel **a**), Race and ethnicity (panel **b**), type of insurance (panel **c**), and rural or urban place of residence (panel **d**)

We plotted predicted probabilities of screening at two years for the 2017 and 2018 cohorts, by sociodemographic groupings (Fig. 1). In the interaction models, we tested whether sociodemographic inequities in screening between the two cohorts were different in 2020 compared to 2019 (Table 3). These models did not indicate statistically-significant differences in the age- or rurality- related inequities. However, decline in screening among women using Medicaid insurance was sharper than for those using private insurance, widening inequities during the pandemic (OR 0.74, *p* = 0.016). Decline in screening for Asian women was also sharper compared to white women (OR 0.76, *p* = 0.026), with Black women showing a similar but non-significant trend (OR 0.79, *p* = 0.060). For all other sociodemographic analyses, there was no evidence of widening inequities during the pandemic.

**Table 3:** Odds ratios (OR) from four multivariable-adjusted logistic regression interaction models assessing sociodemographic inequities in two-year repeat screening for the 2018 cohort compared to the 2017 cohort

	Age*Cohort Interaction				Race*Cohort Interaction		
	OR	CI	*p*		OR	CI	*p*
AgeCat 50–64	Reference			White	Reference		
AgeCat 65–69	0.95	0.83–1.10	0.511	AIAN	2.09	0.77–5.76	0.146
AgeCat 70 +	0.94	0.84–1.05	0.263	Asian	0.76	0.59–0.97	0.026
				Black	0.79	0.62–1.01	0.060
				Hispanic	0.94	0.66–1.34	0.731
	Insurance*Cohort Interaction			Multi	0.93	0.71–1.23	0.615
	OR	CI	*p*	NHOPI	0.68	0.35–1.28	0.237
Commercial	Reference						
Government	0.86	0.53–1.39	0.552		Rural*Cohort Interaction		
Medicaid	0.74	0.58–0.95	0.016		OR	CI	*p*
Medicare	0.97	0.88–1.08	0.631	Urban	Reference		
Selfpay	1.51	0.87–2.59	0.139	Rural	0.89	0.58–1.37	0.615

Only the interaction terms are shown for each model. ORs below 1 indicated greater decline in screening in 2020 compared to 2019, relative to the reference group in each analysis

## Discussion

Our findings add to the growing evidence of the adverse impacts of the coronavirus pandemic on patient access to preventive care. Breast screening on a two-year interval is widely recommended for women 50–74 years [6], because of escalating incidence in this life stage [3]. In this study of women 50 + years of age served by one of the largest health care network in Washington state, we found that only 27% who had received routine breast cancer screening in 2018, presented for screening in 2020, in the midst of the pandemic. This contrasted with an earlier cohort of women screened in 2017, where approximately 37% returned for routine two-year screening in 2019, prior to the pandemic. In both cohorts, some sociodemographic groups were even less likely to adhere to the two-year recommendations, including multi-racial women as well as women residing in rural communities and those on Medicaid and who self-paid for medical care (typically un-insured women).

Our analysis of breast screening data for women 50 years + over the pandemic period is distinct from previous reports, which have assessed the aggregate effect of the pandemic on access-to and use-of breast cancer screening by the general population, irrespective of age-related risk and specific prevention guidelines. Our focus on racial and ethnic minorities and other demographic groups allowed us to further explore potential inequities in the maintenance of cancer screening protocols among vulnerable populations during the months during and after the state-wide shutdown. Moreover, our comparison of two cohorts of women over time allowed us to examine whether known sociodemographic inequities in routine screening varied prior-to and during the pandemic. Our results indicate that although most inequities persisted, several widened during the pandemic.

Health inequities have been a defining feature of the coronavirus pandemic directly and indirectly. Directly, in terms of morbidity and mortality [17], and indirectly, in terms of exposure, and social and economic impacts of the societal response to the pandemic [18]. Our observation that only about 27% women overall adhered to the two-year cancer screening recommendations during the pandemic (compared to 37% of women pre-pandemic) is consistent with the bulk of earlier research demonstrating similar reductions in use of health services broadly [7, 19, 20] and cancer prevention specifically [10–14]. The differential adherence to screening recommendations by race/ethnicity, insurance type, and rural residence we observed may be due the indirect impacts of the pandemic, via containment measures taken by governments.

In Washington State, local and state-wide measures beginning in March 2020 included business and school closures and stay-at-home orders [21]. Many of these measures extended through the end of the year, with phased re-opening of some industries and sectors depending on local conditions. These containment measures dramatically increased unemployment and reduced financial and food security [18, 22, 23], particularly for people who were low- to middle-income or employed in certain occupations prior to the pandemic. In this way the pandemic’s effects on the social determinants of health likely had a disproportionate impact on women from populations that already faced barriers to screening and other cancer preventive services [24–26]. The widening gap between commercially-insured women and those on Medicaid we reported is congruent with this pattern, since Medicaid-insured women are primarily residing in low-income households [27]. Previous research on screening mammography has found that Asian American women were no different from non-Hispanic whites in receiving screening mammograms [25], we observed Asian American women showed a significant decline in screening during the pandemic compared to whites. Further research will be needed to identify the causes of this widening disparity in preventive care.

### Limitations and methodological considerations

A few limitations and methodological aspects of this study should be acknowledged. First, the two cohorts of more 16,391 and 18,197 women were already receiving health care in 2017 and 2018, respectively. As a result, our findings might not reflect women who had limited or no access to care. Related, Washington state, where our study was based, is generally less diverse and more affluent and with lower rates of uninsured residents than other regions of the United States [28]. This may make our findings less generalizable but likely indicate more favorable trends compared to other states with lower incomes and rates of health insurance coverage. Second, our analytic sample assumed a two-year interval between screenings, which was the standard screening schedule widely recommended by public health and medical agencies for women 50 years + with average risk [6]. However, some health care providers may have not recommended this interval to their individual patients, and our methods likely led us to underestimate loss to follow-up screening of some patients were advised to be screened annually. Third, the loss to follow-up screening in 2020, may have reflected moving to different provider rather than loss of screening, which we could not ascertain. Yet the number of patients served by MultiCare, the health care network we studied, (measured as unique patients who completed an encounter with a primary care provider within the health system) did not substantially decrease during the pandemic: 193,174 patients in 2018, 214,305 in 2019 and 211,554 in 2020. Additionally, although there were deaths confirmed among women in both the 2017 and 2018 cohorts, the percentage of deaths in women who did not return for screening in 2019 and 2020 likely did not explain the differences in two-year screening. In the 2017 cohort, 441 or 3.8% of those women who did not present for screening in 2019 were confirmed deceased by 2021, while 263 or 2.2% of women who were screened in 2018 but did not present for screening in 2020 were deceased. This indicates that any change in screening during the pandemic was likely not due to change in total patients served by this health care system or disproportionate deaths in the 2018, cohort.

## Conclusions

For women 50 years and older, routine screening mammography at a minimum of two-year intervals is integral to early detection and increased survival with breast cancer. Our findings indicate that inequities in routine screening prior to the pandemic remained or were amplified during the pandemic. Increasing access to health services and educational programs may help reduce inequities and ensure greater continuity of preventive services generally and during future societal disruptions.

## Data Availability

Clinical data used in this study are not publicly available. Researcher’s may contact MultiCare of Washington to apply for access.
