# Correction to: Racial and socioeconomic inequities in breast cancer screening before and during the COVID-19 pandemic: analysis of two cohorts of women 50 years + 

**DOI:** 10.1007/s12282-022-01361-1

**Published:** 2022-04-30

**Authors:** Pablo Monsivais, Solmaz Amiri, Jeanne Robison, Chaya Pflugeisen, Gordon Kordas, Ofer Amram

**Affiliations:** 1 Department of Nutrition and Exercise Physiology, Elson S. Floyd College of Medicine, Washington State University, 412 E Spokane Falls Blvd, Spokane, WA 99202 USA; 2 Institute for Research and Education To Advance Community Health, Elson S. Floyd College of Medicine, Washington State University, Seattle, WA USA; 3 MultiCare Institute for Research and Innovation, Tacoma, WA 984145 USA; 4 MultiCare Deaconess Cancer and Blood Specialty Centers, Spokane, WA 99204 USA; 5 Department of Medical Education and Clinical Sciences, Elson S. Floyd College of Medicine, Washington State University, Spokane, WA USA; 6 Paul G, Allen School for Global Animal Health, Washington State University, Pullman, WA 99164 USA

## Correction to: Breast Cancer 10.1007/s12282-022-01352-2

In the original publication of the article, the Table 1 caption is wrongly published. The correct caption is given below:

Table 1 Characteristics of two cohorts of women age 50 + Y who had breast cancer screening either in 2017 or 2018, overall and stratified by whether they had a subsequent screening two years following.

